# Annual Meeting of the Italian Surgical Society

**Published:** 1887-05

**Authors:** A. Lagorio

**Affiliations:** Chiavari, Italy


### FOREIGN CORRESPONDENCE.

### ANNUAL MEETING OF THE ITALIAN SURGICAL SOCIETY.

CHIAVARI, ITALY, April 15th, 1887.

*Special Correspondence of The Chicago Medical Journal and Examiner.*

The fourth annual meeting of the Italian Surgical Society was held in Genoa on the 4th, 5th, 6th and 7th of this month. The meeting was largely attended; most of the prominent surgeons of the state were present. Every member reported his subject orally, saving, by so doing, a great deal of time. This enabled the members to get through a large amount of work, and devote more time to the discussions.

The meeting was opened by the President of the Executive Committee, Professor Durante, of Rome. He eulogized in a few well-chosen words the memory of the late Professor F. Margary, describing the progressive, loyal and sincere character of the deceased. He then presented the annual financial report of the society; after which he gave the chair to Professor Azzio Caselli, President of the Society, who welcomed in the name of Genoa the members present. The presentation and discussion of subjects were then proceeded with.

Professor Ceccherelli of Parma, who was announced to lead the discussion of post-operative fever, entertained the society instead on the operation of supra-pubic cystotomy for the relief of vesical tumors. He had performed this operation on a man 70 years old, for a malignant growth of the bladder, with success. He spoke at length on the diagnosis of these cases, especially regarding hæmorrhage as a symptom.

Professor Novaro, of Siena, read a very interesting communication on an experimental operation performed in January on a dog, for the insertion of both ureters in the rectum. The operation was successful and the dog is now living and doing well. This operation was partially, and without good success, attempted by Gluck and Zeller of Berlin. Bardenhauer of Cologne succeeded with only one of the ureters. Professor Novaro thought that in cancerous tumors of the bladder the complcte extirpation of the bladder ought to be performed in two operations, reserving the exportative for the last. Scooping such a fragile organ only resulted in harm; moreover, only arrested the hæmorrhage for a few days. The members applauded Professor Novaro for his successful result.

Professor Ruggi, of Bologna, entertained the society on several laparotomies performed last year with good success.

Dr. Lampugnani, of Turin, described a modification which he had adopted in the decapitation of the femur for the relief of congenital dislocation of the hip joint.

Professor Ceccherelli reported a number of experiments on the surgery of the stomach. He also related two cases of craniotomy for the relief of tumors.

Professor De Rossi, of Rome, reported several malignant tumors of the mastoid process. This communication gave rise to a lively discussion, in which Professors Bottini, Novaro, D'Antona, Durante and others joined.

Professor Paci, of Pisa, reported a total removal of the scapula and three partial ones.

Professor D'Antona, of Naples, reported several cases of ascites treated with drainage. He also reported several laparotomies.

Professor Paci referred to an old traumatic iliac dislocation of the femur treated with the femoral decapitation.

Professor Durante spoke at length on tuberculosis and pseudo-tuberculosis of bones and synovial membranes. He called it an exaggeration to believe all chronic articular lesions tuberculosis. There are many inflammatory forms which may be mistaken for tubercular products. He presented a number of anatomical and clinical facts to prove his assertions.

Professor De Rossi called attention to the good results obtained by him in the treatment of osseous and articular tuberculosis with sea-baths. He showed photographs of many patients cured without operative procedures.

Professor Paci related the history of three cases of club feet. Two of these were cured with the removal of the astragalus, and the other with the resection of the nervous branches entangled in the ankle-joint from an epiphyseal separation.

Dr. Lampugnani spoke on the treatment of varus-equinus, and in particular on the extirpation of the astragalus. These subjects were discussed by Drs. Bottini, Novaro, Durante, Ceci, Postensky, D'Antona and others.

Dr. Tricomi, of Naples, made known his clinical and experimental researches on some suppurative processes. He had always found present the staphylococcus aureus, never the albus. Professor Ceci on the contrary had frequently seen the albus.

Dr. Tricomi also spoke on the antiseptic properties of iodol and iodoform. He had found iodol far superior as an antiseptic.

Professor Ruggi presented to the society a new cystotome. He had invented it for those who were not familiar with lithotomy; with it they could operate without fear of failing. The ingenious instrument consisted of a staff, in which was concealed a trocar. The staff was introduced in the bladder, and by turning a screw the trocar could be made to cut from within outwards. The instrument was not favorably received by the members. Professor Novaro thought the instrument was too complicated. Our surgical instruments ought to be simplified. Such instruments as the one in question would spoil surgery.

Professor D'Antona said that lithotomy had now reached such perfection and facility that we ought to consider as superfluous any other new method of performing it. Professor Ruggi defended his cystotome. He had not invented it for teachers of surgery. He invited the members to the dead-house, where he would illustrate its working on the cadaver.

Professor Durante made a report of four cases in which he showed the utility of an artificial anus, as a preliminary treatment in cancerous condition of the rectum. He also reported a case in which he had ligated the innominate artery for the relief of a subclavian aneurism with perfect success. He had used catgut, to which he attributed a great share of his success. He said that this case was the nineteenth on record, and, with that of Dr. Smythe of New Orleans, the second successful one. The Professor was highly complimented by all present.

Professor Novaro reported a successful case of cholecystotomy—the first one on record in Italy. A patient, female, 43 years old, suffered several times from bilious colic. When brought to the hospital she was suffering with pain in the region of the liver, was feverish, jaundiced and suffered from vomiting. Bile feces were wanting. A tumor was detected over the liver, which was immediately diagnosed as an enlarged gall bladder. On investigation pus, bile and mucus were obtained. The patient did not improve. The operation of cholecystotomy was decided upon. An incision was made over the tumor, the abdominal wall cut through, the gall bladder found, incised, and stitched to the sides of the external wound. A large stone was found lodged in the ductus, and was removed with great difficulty. The wound was dressed, but a fistulous opening remained, through which bile continued to be discharged. After three months, the fistula showed no tendency to heal, and the edges were touched with lunar caustic and made to granulate, and gradually it healed. The patient is now enjoying good health, and has had no sign of her former trouble.

Dr. Bonanno, of Rome, reported a case of an extra-uterine pregnancy, in which the sac and the fœtus were situated on the anterior portion of the body of the uterus. How the ovum got there he could not explain. The patient, in the sixth month of pregnancy, feeling no longer the movements of the fœtus, consulted him, and he, after a careful examination, made a diagnosis of death of the fœtus. He performed laparotomy, removed the dead fœtus and the sac, and the patient made a good recovery.

Professor Ferrari, of Parma, spoke on the ætiology of tetanus. From several experiments made in his clinic and laboratory he thought tetanus to be due to a specific microbe (a staphylococcus); he was continuing the experiments, and he would at the next annual meeting make known his final conclusions. Professors Ceci, Tricomi, Berti, and others discussed this subject.

Professor Caselli, of Genoa, spoke on the subject of tracheotomy, and showed some modifications which he had made in the instrument. He modified the tenaculum in having it grooved on the convex side, so that, with the help of a grooved director, he could be enabled to quickly slide the tube on to the trachea. The quicker one can put the tube in the wind-pipe the better will be the result of the operation, for the secret of success lies in preventing any blood from entering the trachea. He produced statistics of 132 operations with 53 recoveries, while since his modification he has had 13 recoveries in 18 operations, a remarkably good showing. During the discussion I was very much surprised at not hearing any allusion made to the process of intubation of the larynx.

Professors Ruggi, Lampiasi, Rubino, Crespi-Morini, and D'Antona, each presented photographs of patients for whom they had performed Estländer's operation for the relief of empyæma.

Professor Ceci related a case in which he had trephined the skull of a patient brought to the hospital in a hemiplegic and comatose condition, resulting in complete recovery. He also related several successful plastic operations. He also showed to the society a patient from whom he had last year removed the spleen. She has since been enjoying good health, and has increased 7½ kilograms in weight. On examining her I noticed that the thyroid gland on the right side was becoming enlarged, as were also both tonsils. I leave it to the physiologists to explain this condition.

Professor Ceci, also showed another patient from whom he had removed the upper half of the scapula, leaving the periosteum. The whole bone was reproduced, together with the spine and processes.

Dr. Celsus Motta, of Genoa, presented a patient in whom he had preformed a resection of the knee-joint according to

Caselli's method. This method has given excellent results in his clinic in Genoa; and, as it is simple and easy, I will endeavor to briefly describe it:

The articulation is opened with a semi-lunar incision, which begins and terminates four centimeters above the condyles, cutting the ligamentum patellæ at its middle. Having dissected and raised the flap so formed, we expose the femoral epiphysis which is resected. This resection is performed with the saw, and is made beginning two centimeters above the anterior inter-condyloid notch, and terminating at the insertion of the crucial ligaments. With this section we expose, as we readily understand, not only the spongy substance of the epiphyses, but also the epiphyseal cartilage and the spongy substance of the inferior extremity of the diaphysis of the femur. It permits us, therefore, to minutely examine this part, and if any diseased points are found they can be removed with Volkmann's spoons. For the tibia it is sufficient ordinarily, as has been noted by König, to perform a section of the cartilaginous meniscus, or of the most superficial layers of the epiphyses. This can easily be done with the scalpel. Finally, to complete the resection, we remove, where it is possible, the articular capsule; if not possible, we excise the synovial membrane and scoop accurately with Volkmann's spoons. This operative process has the great advantage of permitting the removal of the morbid portion of the spongy substance of the femur, preserving most of the epiphyseal cartilages. The crucial ligaments are not touched, and therefore we preserve the normal anatomical relations of the extremities of the bones, while in Volkmann's process the bony ends are completely dissected, and by so doing the whole articular cavity is exposed.

Professor Caselli's method does not alter the length of the femur, nor of the tibia, nor their normal relations; therefore there is no shortening.

Dr. Caneva, of Genoa, followed with an excellent paper on the transplantation of the tendonous insertions in the bones.

Professor Caselli made a report of several removals of intracranial tumors. He also related a case of extrophy of the bladder.

Professor Bassini, of Padua, described a new operation for the radical cure of inguinal hernia, practiced by him with success; it was unanimously approved. The learned Professor was made the recipient of many complimentary remarks.

Professor Bonora entertained the society on the subject of ovariotomy, reporting a number of cases. He also reported a successful operation of a resection of a piece of gangrenous intestine.

Dr. Muguai, of Rome, reported many resections of the knee-joint.

Professor Roth spoke on the supra-pubic operation for the extraction of vesical calculi.

The election of officers for the ensuing year then occurred, with the following result: Professor Antonio D'Antona, president; Professors Bottini and Corradi, vice-presidents. Naples was chosen for the next place of meeting.

A. LAGORIO, M.D.

